# The YUCCA-Auxin-WOX11 Module Controls Crown Root Development in Rice

**DOI:** 10.3389/fpls.2018.00523

**Published:** 2018-04-23

**Authors:** Tao Zhang, Ruonan Li, Jialing Xing, Lang Yan, Rongchen Wang, Yunde Zhao

**Affiliations:** ^1^National Key Laboratory of Crop Genetic Improvement and National Center of Plant Gene Research (Wuhan), Huazhong Agricultural University, Wuhan, China; ^2^Section of Cell and Developmental Biology, University of California, San Diego, San Diego, CA, United States

**Keywords:** auxin, WOX11, crown root, YUCCA, TAA

## Abstract

A well-developed root system in rice and other crops can ensure plants to efficiently absorb nutrients and water. Auxin is a key regulator for various aspect of root development, but the detailed molecular mechanisms by which auxin controls crown root development in rice are not understood. We show that overexpression of a *YUC* gene, which encodes the rate-limiting enzyme in auxin biosynthesis, causes massive proliferation of crown roots. On the other hand, we find that disruption of *TAA1*, which functions upstream of *YUC* genes, greatly reduces crown root development. We find that *YUC* overexpression-induced crown root proliferation requires the presence of the transcription factor WOX11. Moreover, the crown rootless phenotype of *taa1* mutants was partially rescued by overexpression of *WOX11*. Furthermore, we show that *WOX11* expression is induced in *OsYUC1* overexpression lines, but is repressed in the *taa1* mutants. Our results indicate that auxin synthesized by the TAA/YUC pathway is necessary and sufficient for crown root development in rice. Auxin activates *WOX11* transcription, which subsequently drives crown root initiation and development, establishing the YUC-Auxin-WOX11 module for crown root development in rice.

## Introduction

Roots determine the amount of nutrients and water available for plant growth and development, direct impacting yield and other agriculturally important traits. Rice root system consists of seminal roots and postembryonic shoot-borne crown roots with lateral roots branching off from both (Mai et al., 2014). Because of its agronomic importance, rice root system has been studied extensively using both genetic and genomic approaches. The emerging picture is that auxin plays an essential role in almost every aspect of rice root growth and development. Disruption of auxin biosynthesis, metabolism, transport, or signaling has a profound impact on rice root development.

Genetic screens for mutants that display altered patterns and/or morphology of root systems identified multiple loci (Mai et al., 2014). Molecular cloning and characterization of the rice root mutants clearly demonstrated the essential roles of auxin in root development. For example, gain-of-function mutations in the domain II of *OsIAA11* and *OsIAA13*, which encodes negative regulators of auxin signaling, abolish lateral development (Kitomi et al., 2012; Zhu et al., 2012). Gain-of-function mutations in *OsIAA23* lead to a dramatic reduction of crown roots and lateral roots (Jun et al., 2011). Moreover, *OsIAA23* is required for QC maintenance (Jun et al., 2011). Other auxin signaling components such as *OsTIR1/AFB2* (Xia et al., 2012)*, OsCAND1* (Wang et al., 2011), *OsCYP2* (Kang et al., 2013)*, LATERAL ROOTLESS2 (LRT2)* (Jing et al., 2015) are also required for crown root and lateral development. Forward genetic screens have isolated six *crown rootless* (*crl*) mutants, which either did not develop any crown roots or had dramatically reduced number of crown roots (Inukai et al., 2001). *CRL1* encodes the OsLBD3-2, which is transcriptionally regulated by the Auxin Response Factor 16, suggesting that CRL1 is also part of the auxin regulated network required for crown root development (Inukai et al., 2001; Coudert et al., 2015). The *crl2* and *crl3* mutants are defective in crown root primordia development and cell elongation (Inukai et al., 2001; Kitomi et al., 2008), but the molecular identities of *CRL2* and *CRL3* have not been determined. The *crl4* phenotypes were caused by a mutation in the *GNOM1* gene, which encodes a ADP-ribosylation factor, and which is implicated in trafficking of the auxin efflux carrier PIN-FORMED (PIN) proteins, suggesting that polar auxin transport is also required for rice root development (Liu et al., 2009). Overexpression of *OsPID*, which was proposed as a regulator of PIN polarity, also affects root development (Morita and Kyozuka, 2007). Other auxin transport-related genes including *OsPIN1* (Xu et al., 2005), *OsPIN2* (Chen et al., 2012), and *OsAUX1* (Zhao H. et al., 2015) have been implicated in crown root development as well. *CRL5* encodes a member of the large AP2/ERF transcription factor family (Kitomi et al., 2011). The *crl5* mutant produced fewer crown roots and displayed impaired initiation of crown root primordia (Kitomi et al., 2011). *CRL5* is also part of the auxin regulated network because exogenous auxin treatment induced *CRL5* expression without *de novo* protein biosynthesis. Auxin-induced *CRL5* expression requires the degradation of AUX/IAA proteins. OsARF1 binds to the *CRL5* promoter, and *CRL5* controls the cytokinin signaling pathway via type-A response regulators (ARRs) (Kitomi et al., 2011). *CRL6* encodes a member of the large chromodomain, helicase/ATPase, and DNA-binding domain (CHD) family protein (Wang et al., 2016). *CRL6* influences crown root formation by regulating primordial initiation and development. It was shown that the expressions of *OsIAA* genes were down-regulated in *crl6*, linking *CRL6* to auxin regulatory network (Wang et al., 2016).

Auxin is mainly synthesized by the TAA/YUC pathway, which is highly conserved throughout the plant kingdom (Zhao, 2012). TAA aminotransferases convert Trp to Indole-3-pyruvate (IPA), which subsequently undergoes oxidative decarboxylation catalyzed by the YUC flavin monooxygenases to produce IAA (Mashiguchi et al., 2011; Won et al., 2011). It was shown that auxin synthesized by the TAA/YUC pathway plays critical roles in root development in Arabidopsis (Stepanova et al., 2008; Chen et al., 2014). Disruption of various combinations of *YUC* genes and/or *TAA* genes can cause moderate to very severe root defects in Arabidopsis. For example, the *taa1 tar1 tar2* triple mutants in Arabidopsis fail to make root meristem during embryogenesis (Stepanova et al., 2008). Similar phenotypes were observed in *yuc1 yuc4 yuc10 yuc11* quadruple mutants (Cheng et al., 2007). Some other *yuc* combinations in Arabidopsis such as *yuc3 yuc5 yuc7 yuc8 yuc9* quintuple mutants (*yucQ)* have very short and agravitropic roots (Chen et al., 2014). In rice, the *taa1* mutants, also known as *fib1(fish bone 1)* display pleotropic phenotypes including agravitropic roots, long seminal roots, few crown roots, and a lack of lateral roots (Yoshikawa et al., 2014). Other TAA homologs in rice such as *OsTAR1* is likely also involved in auxin biosynthesis (Kakei et al., 2017). Overexpression of *OsYUC1* increased IAA levels and led to characteristic auxin overproduction phenotypes including thick hairy roots, ectopic crown roots developed from elongated node, and defective leaf growth (Yamamoto et al., 2007). Inhibition of *OsYUC1* expression by antisense construct leads to severe shoot dwarfism and defective root formation (Yamamoto et al., 2007). However *OsYUC4* RNAi lines did not displayed abnormal phenotype (Yamamoto et al., 2007). Both the *OsCOW1* (*CONSTITUTIVELY WILTED 1*) and *OsNAL7* (*NARROW LEAF 7*) are the *OsYUC8* gene (Woo et al., 2007; Fujino et al., 2008). The *Osyuc8* mutants greatly reduced the amount of roots (Woo et al., 2007; Fujino et al., 2008). It has been evident that auxin synthesized by the TAA/YUC pathway plays important roles in rice root development.

Besides auxin, the transcription factor *WOX11* appears to play a paramountly important role in root development (Zhao et al., 2009). Crown root development is inhibited in the loss-of-function *wox11* mutants, whereas overexpression of *WOX11* stimulates crown root growth and the development of crown roots from the upper stem nodes (Zhao et al., 2009). It was shown that *WOX11* interacts with ERF3 and binds to the RR2 promoter to directly regulate the crown root development (Zhao Y. et al., 2015). *WOX11* is also involved in lateral root initiation, root hair formation, and abiotic stress-responsive development (Zhao Y. et al., 2015; Cheng et al., 2016). Furthermore, it has been suggested that WOX11 can recruit ADA2-GCN5 histone acetyltransferase module to activate downstream target genes in crown root development (Zhou et al., 2017). In Arabidopsis, WOX11 was induced by exogenous IAA application and it controls the first-step of cell fate transition during callus initiation (Liu et al., 2014; Hu and Xu, 2016). WOX11 also mediated the primary root development by regulating the *WOX5/7* expression with or without auxin induction (Sheng et al., 2017).

In this paper, we investigate the relationship between auxin and WOX11, the two important regulators of crown root development in rice. We show that overexpression of the *YUC* genes leads to massive over proliferation of crown roots. However, in the absence of *WOX11*, overexpression of *YUC* genes did not stimulate crown root development. On the other hand, overexpression of *WOX11* in the *taa1/fib1* mutant background, which fails to produce crown roots, restored the crown root development. Our results demonstrate that auxin synthesized by the TAA/YUC pathway is necessary and sufficient for crown root initiation and development. Moreover, we show that WOX11 functions downstream TAA/YUC pathway and that auxin-induced crown root development is largely controlled by WOX11.

## Materials and Methods

### Phylogenetic Analysis of OsYUCs and AtYUCs and Structural Analysis of *OsYUC* Genes

Multiple-alignment was performed using Clustal Omega (McWilliam et al., 2013) and the resulting sequence alignments were then used to construct the unrooted phylogenetic tree by the neighbor joining method with a bootstrap analysis of 1000 replicates using MEGA 7.0 (Kumar et al., 2016). The exon-intron structure of each *OsYUC* gene was identified by using the Gene Structure Display Server (Hu et al., 2015).

### Genotyping of *oswox11-1* and *ostaa1/fib1*

The mutant *oswox11-1* was obtained from Zhao et al. (2009). The insertions were confirmed by PCR using *WOX11*-specific primers WOX11-F2 and WOX11-R2 and the T-DNA left side primer L2. The primer sequences were shown in Supplementary Table S2.

To genotype the *taa1/fib1* mutants generated by CRISPR, we amplified about 500 bp DNA-fragment that covers the CRISPR target sequence using the primers Taa1-seqF/Taa1-seqR (Supplementary Table S2). The PCR products were sequenced directly using the Taa1-F primer.

### Cloning of the DNA Constructs for *OsYUCs* and *OsWOX11* Overexpression

The cDNAs of *OsYUC1*, *OsYUC5*, *OsYUC6*, *OsYUC7*, and *OsWOX11* were amplified from the rice cultivar Zhonghua 11 (ZH11) and then cloned into the pCAMBIA1301U-HPT. The cDNAs were placed under the control of the maize *Ubiquitin* promoter. We also cloned the genomic fragments of *OsYUC3*, *OsYUC4*, *OsYUC8*, *OsYUC10*, *OsYUC11*, and *OsYUC14* from the rice cultivar Zhonghua 11 (ZH11) into the pCAMBIA1301U-HPT to overexpress them.

### Construct CRISPR Mutants of Rice *TAA1*

The binary vector pCXUN (Chen et al., 2009) was used for making the CRISPR/Cas9 backbone vector pCXUN-Cas9 (He et al., 2017). Specifically, it was constructed by inserting the rice codon-optimized Cas9 between the two *XcmI* sites under control of the maize *UBIQUITIN* promoter. The *TAA1*-specific guide RNA was produced by the rice *U3* promoter.

### Rice Transformation

Rice cultivar ZH11 (*Oryza sativa L.* ssp. *japonica*) was obtained from the rice collection of the National Key Laboratory of Crop Genetic Improvement, Wuhan, China. ZH11 was transformed via *Agrobacterium tumefaciens* (EHA105)-mediated callus transformation as previously described (Hiei et al., 1994). Overexpressing constructs of *OsYUC1*, *OsYUC3*, *OsYUC4*, *OsYUC5*, *OsYUC6*, *OsYUC7*, *OsYUC8, OsYUC10*, *OsYUC11*, and *OsYUC14* were transformed into ZH11. The *OsYUC1* overexpression construct was also transformed into Hwa (used as wild type variety) and *oswox11-1* mutant. The *OsWOX11* overexpression construct was transformed into *ostaa1fib1* mutant calli.

### RNA Isolation and RT-PCR Analysis

Total RNAs were isolated using TRIzol reagent (Invitrogen). Complementary DNAs were made by reverse-transcription according to the manufacturer’s instructions (Invitrogen). RT-qPCRs were performed using gene-specific primers (Supplementary Table S2) and SYBR Premix Ex-Taq on a real-time PCR 7500 system (Applied Biosystems). Data were collected using the ABI PRISM 7500 sequence detection system following the manufacturer’s instruction. The rice *ACTIN1* gene was used as the internal control. At least three biological replicates and three technical repeats were conducted.

### Auxin Measurements

The extraction and measurement of auxin in rice were conducted using a previously reported method (Liu et al., 2012).

## Results

### Induction of Ectopic Crown Root Development by Overexpressing of *OsYUC1*

**FIGURE 1:**
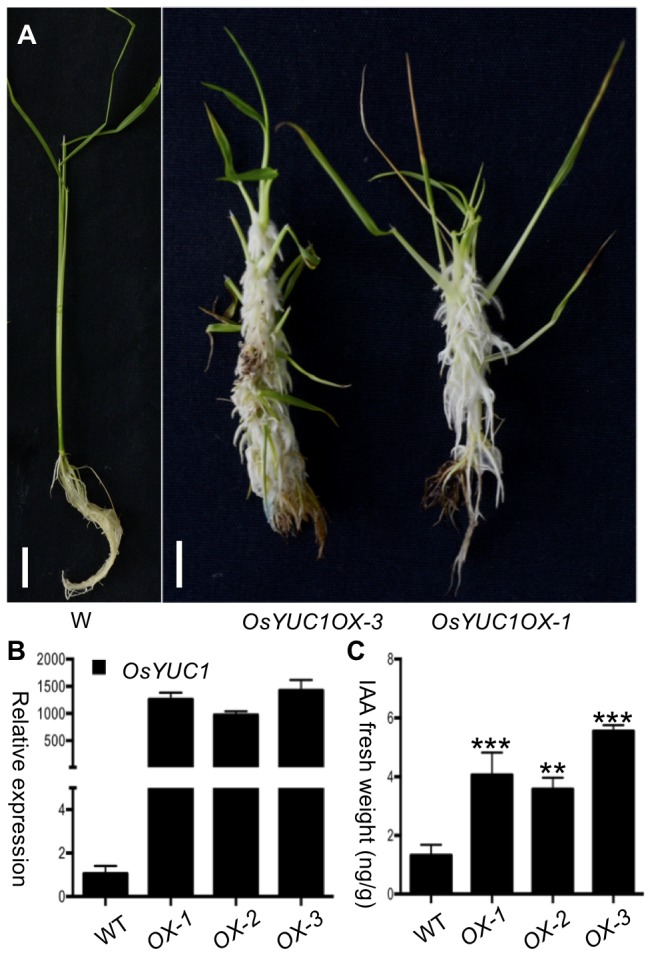
Overexpression of *OsYUC1* promotes crown root proliferation and auxin overproduction. **(A)** Massive crown root proliferation was observed when *OsYUC1* was overexpressed (the right two plants). The overexpression lines initiated many crown roots from the shoots. The crown roots also had more and longer root hairs compared to the wild type plant (left). *OsYUC1OX* refers to *OsYUC1* overexpression under the control of the strong maize *UBIQUITIN* promoter. *OsYUC1OX-1 and OsYUC1OX-3* were two independent TO plants. Bar = 1 cm. **(B)** Relative transcript levels (folds) of *OsYUC1* in the independent overexpression lines. *OX* refers to *OsYUC1* overexpression. WT refers to WT. Note that expression levels in the OX lines were increased hundreds fold. **(C)** Comparison of IAA contents in whole seedlings of WT and *OsYUC1* overexpression tines. The data are presented as mean ±SD (*n* = 3). ^∗^*P* < 0.05, ^∗∗^*P* < 0.01, ^∗∗∗^*P* < 0.001 (Student’s *t*-test).

*OsYUC1* was previously implicated in auxin biosynthesis. It was reported that abnormal roots were produced at the regeneration stage and rooting stage when *OsYUC1* overexpression construct was transformed into rice calli through Agrobacteria-mediated transformation (Yamamoto et al., 2007). We overexpressed *OsYUC1* in rice as part of our effort to understand auxin biosynthesis in rice. Although it is known that overexpression of *OsYUC1* stimulates root development, we were still surprised by the massive proliferation of the root system induced by elevated *OsYUC1* expression (**Figure 1A**). The *OsYUC1* overexpression lines initiated massive number of roots from mesocotyls, leaf sheath, and other shoot parts. The lush root hairs and adventitious roots/crown roots cover almost the entire shoots (**Figure 1A**). The expression levels of *OsYUC1* correlate well with the severity of the phenotypes observed in the *OsYUC1* overexpression lines (**Figure 1B**). We further analyzed the IAA concentrations in some of the *OsYUC1* overexpression lines. As expected, overexpression of *OsYUC1* led to increased auxin concentrations (**Figure 1C**).

### Rice Has 14 *YUC* Genes for Auxin Biosynthesis

**FIGURE 2:**
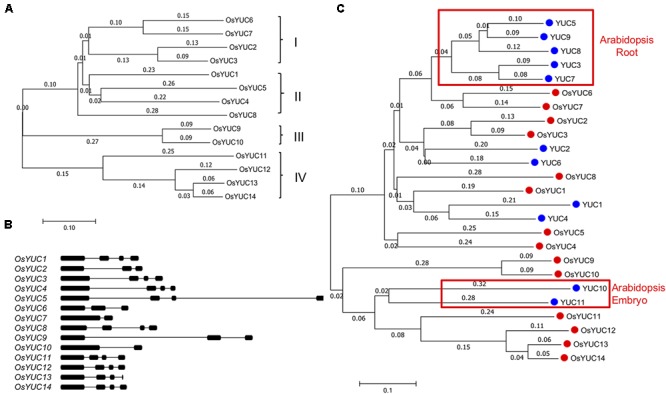
Identification and analysis of putative rice *YUC* genes. **(A)** Phylogenic analysis of OsYUCs. The phylogenetic trees were constructed using Mega7.0 program. Based on the analysis, OsYUCs were divided into four sub-groups. **(B)** Gene structures of the *OsYUCs.* Thin lines represent introns, dark bars refer to exons. **(C)** Phylogenic comparison of rice YUCs and Arabidopsis YUCs. Note that Arabidopsis appears to have more root YUCs whereas rice seems to have expanded embryo YUCs.

In order to further study the roles of YUC-mediated auxin biosynthesis in root development, we conducted *in silico* analyses of *YUC* genes in rice and compared them with the Arabidopsis *YUC* genes. Previous bioinformatics analyses identified 7 *YUC* genes in rice (Yamamoto et al., 2007), which can be divided into four sub-groups (**Figure 2A**). We used the Arabidopsis YUC1 protein sequence (AtYUC1) as the query for phylogenetic analysis of *YUC*s in rice and identified 14 *OsYUC* genes that share significant homology with the *AtYUC1* (**Figure 2A**). We can divide the OsYUCs into four groups as well (**Figure 2A**). All of the putative OsYUC enzymes contain the conserved motifs for binding FAD and NADPH cofactors (Supplementary Figure S1) (Hou et al., 2011), suggesting that they are probably functional flavin monooxygenases. All of the *OsYUC* genes have several introns with variable lengths (**Figure 2B**). In contrast, some of the Arabidopsis *YUC* genes such as *YUC8* and *YUC5* do not have any introns (Chen et al., 2014).

We investigated the phylogenetic relationship between rice and Arabidopsis *YUC* genes by comparing the full-length protein sequences (**Figure 2C**). We had two interesting observations: (1) Rice appeared to have dramatically reduced the *YUC* genes grouped to the Arabidopsis root *YUC* genes. In Arabidopsis, five *YUC* genes (*YUC3*, *YUC5*, *YUC7*, *YUC8*, and *YUC9*) were shown to play important roles in Arabidopsis root development (Chen et al., 2014). Rice only has the *OsYUC6* and *OsYUC7* that belong to this group. (2) Rice has expanded the *YUC* genes related to embryogenesis and endosperm development. Arabidopsis *YUC10* and *YUC11* along with *YUC1* and *YUC4* are required for embryogenesis (Cheng et al., 2006). Rice has six *YUC* genes that are closely related to *AtYUC10* and *AtYUC11* (**Figure 2C**).

### Overexpression of Most *OsYUC* Genes Leads to Auxin Overproduction

**FIGURE 3:**
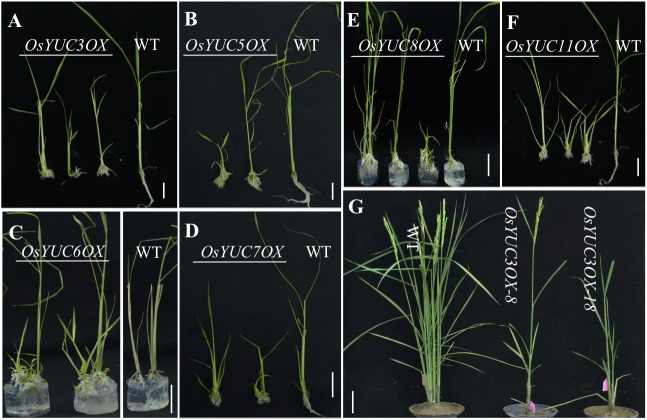
Effects of overexpression of *OsYUC* genes on rice development. **(A)** Overexpression of the *OsYUC3* leads to short seminal root and more crown roots. The shoots of the overexpression lines (left three plants) appear shorter as well. Similar phenotypes were also observed when *OsYUC5* **(B)**, *OsYUC6* **(C)**, *OsYUC7* **(D)**, *OsYUC8* **(E)**, and *OsYUC11* **(F)** were overexpressed. All of the *OsYUC* overexpression lines showed ectopic crown roots. *OsYUCsOX* refers to *OsYUCs* overexpression under the control of the strong maize *UBIQUITIN* promoter. Bar = 3 cm. **(G)** The mature stage plants of WT and *OsYUC3* overexpression lines. Very few tillers were developed in the overexpression lines (the right two plants). *OsYUC3OX-8 and OsYUC3OX-18* were two independent T0 plants. Bar = 10 cm.

To functionally characterize the *OsYUC* genes in rice, we tried to overexpress all of the *OsYUC* genes (either cDNA or genomic sequences) using the strong maize *UBIQUITIN* promoter. Overexpression of any *OsYUC* genes except four caused the obvious auxin overproduction phenotypes. The roles of *OsYUC2*, *OsYUC9*, *OsYUC12*, and *OsYUC13* in auxin biosynthesis have not been experimentally determined yet because we ran into some difficulties in generating their overexpression constructs. Nevertheless, we showed that at least 10 *YUC* genes in rice had the capacity to synthesize auxin. Moreover, overexpression of the *YUC* genes resulted in very similar phenotypes. Representative phenotypes of overexpression of *OsYUC1*, *OsYUC3*, *OsYUC5*, *OsYUC6*, *OsYUC7*, *OsYUC8*, and *OsYUC11* were shown (**Figures 1**, **3**). Overall, overexpression of *OsYUC* genes caused the development of ectopic adventitious root, shortened seminal roots, and over proliferation of root hairs (**Figures 1**, **3**). For example, overexpression of *OsYUC8* led to phenotypes very similar to those observed in *OsYUC1* overexpression.

The strong *OsYUC* overexpression lines died and never produced any seeds. Some moderately overexpression lines were able to reach to adult stage and produce some seeds. The relatively weaker *OsYUC* overexpression lines developed just slightly increased number of crown roots, but the lines also had dramatically reduced tiller numbers. Sometimes, only one single tiller was produced (**Figure 3G**).

### Auxin Synthesized by the TAA/YUC Pathway Is Required for Crown Root Development

**FIGURE 4:**
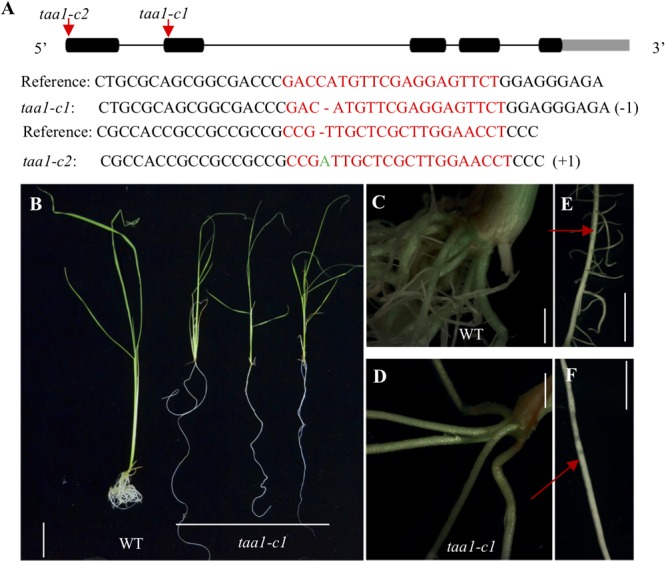
Generation of *CRISPR* mutations in the *TAA1* gene in rice and characterization of the root defects of the *taa1* mutants. **(A)** Two independent alleles of *taa* mutants (*taa1-c1* and *taa1-c2*, c stands for CRISPR) were generated using CRISPR/Cas9 gene-editing technology as described in the Materials and Methods. The mutations were located inside the *TAA1/FIB1* target sequence. The arrows indicate CRISPR/Cas9 target sites, which were located inside the first and the second exon, respectively. The *taa1-c1* had one 1 bp deletion and the *taa1-c2* contained 1 bp insertion. **(B)** Comparison of the seedlings of *taa1-c1* homozygous mutants with WT. The *taa1-c1* mutant had longer seminal root and fewer crown roots compared to WT. Bar = 3 cm. **(C–D)** The detailed root images of WT and *taa1-c1* homozygous mutants, respectively. The *taa1-c1* mutant failed to develop crown roots. Bar = 0.2 cm. WT plants developed lateral roots **(E)** whereas *taa1-c1* did not have lateral roots **(F)**. Bar = 0.5 cm.

Because of the existence of 14 *YUC* genes in rice and some of which are likely have redundant functions, it is difficult to study loss-of-function *yuc* mutants in rice. To generate partial auxin deficient mutants in rice, we turned to the *TAA1/FIB* genes in rice, which have less redundancy and which function upstream of *YUC* (Yoshikawa et al., 2014). Disruption of the *OsTAA1* gene caused pleiotropic phenotypes and decreased IAA content by half compared to wild type (Yoshikawa et al., 2014). We generated *taa1/fib1* mutants using the CRISPR/Cas9 gene editing technology in order to study the roles of auxin in root development (**Figure 4A**). The *taa1/fib1* homozygous mutants showed phenotypes similar to those reported previously (Yoshikawa et al., 2014). The *taa1/fib1* showed characteristic auxin deficient phenotypes including severe dwarf, fewer and smoother crown roots with few lateral roots and longer seminal roots compared to wild type (**Figures 4B–F**). Our results demonstrate that auxin synthesized by the TAA/YUC pathway is required for normal root development in rice.

### Overexpression of *OsYUC* Genes Leads to Transcriptional Activation of WOX11

**FIGURE 5:**
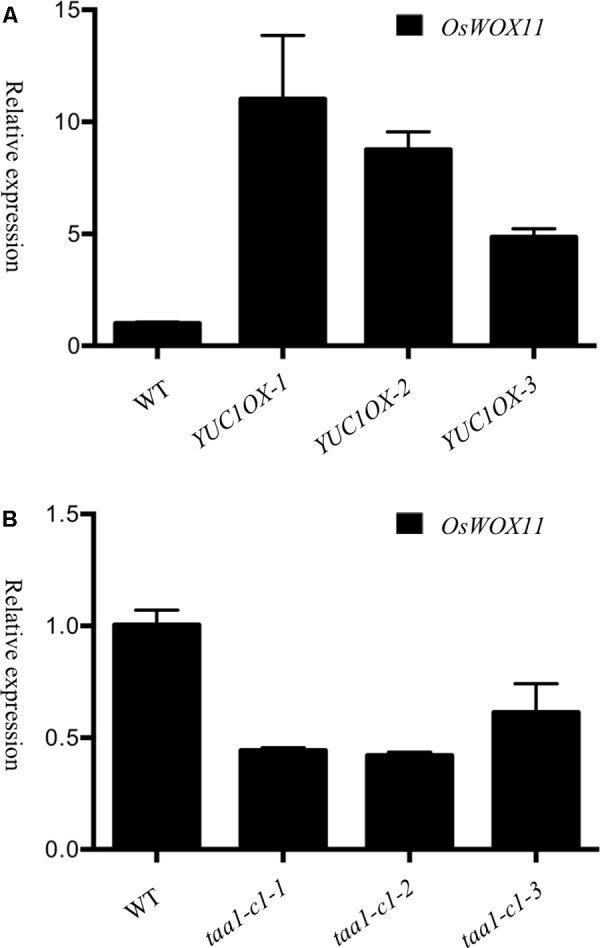
*WOX11* expression is regulated by the TAA/YUC auxin biosynthesis pathway. **(A)** Overexpression of *OsYUC1* induced *WOX11* expression. The relative expression levels of *WOX11* from three independent lines were shown. The *OsYUC1* expression levels and IAA concentrations of’ the three lines were shown in **Figure 1B**. **(B)** *OsWOX11* expression was repressed in *taa* mutants. Relative transcript levels (fold) *of OsWOX11* were decreased in the *taa1-cl* homozygous mutants, *taa1-cl-1, taa1-cl-2* and *taal-cI-3* were individual plants.

Development of ectopic crown roots in the *OsYUC* overexpression lines (**Figures 1**, **3**) resembled the phenotypes observed in the *OsWOX11* overexpression lines (Zhao et al., 2009), though the *OsWOX11* overexpression phenotypes were much weaker. We hypothesized that auxin produced by YUCs might up-regulate *WOX11* expression, consequently stimulating root development. It was previously reported that auxin treatments induced *WOX11* expression in Arabidopsis (Liu et al., 2014). We determined the transcript levels of rice *WOX11* in the *OsYUC1* overexpression lines (**Figure 5A**). Overexpression of *OsYUC1* led to significant increases of *WOX11* transcripts, suggesting a regulatory module of *YUC-Auxin-WOX11* in rice root development.

We also analyzed the transcript levels of *OsWOX11* in *taa1/fib1* mutants, which contain much less auxin compared to wild type (Yoshikawa et al., 2014). If *WOX11* is indeed regulated by auxin at transcription level, we would expect that the expression levels of *WOX11* would be much reduced. Indeed, *OsWOX11* expression were significantly reduced in our *taa1/fib1* mutants (**Figure 5B**).

### Over-Proliferation of Ectopic Roots in *OsYUC1* Overexpression Lines Is Abolished in the *wox11-1* Mutant

**FIGURE 6:**
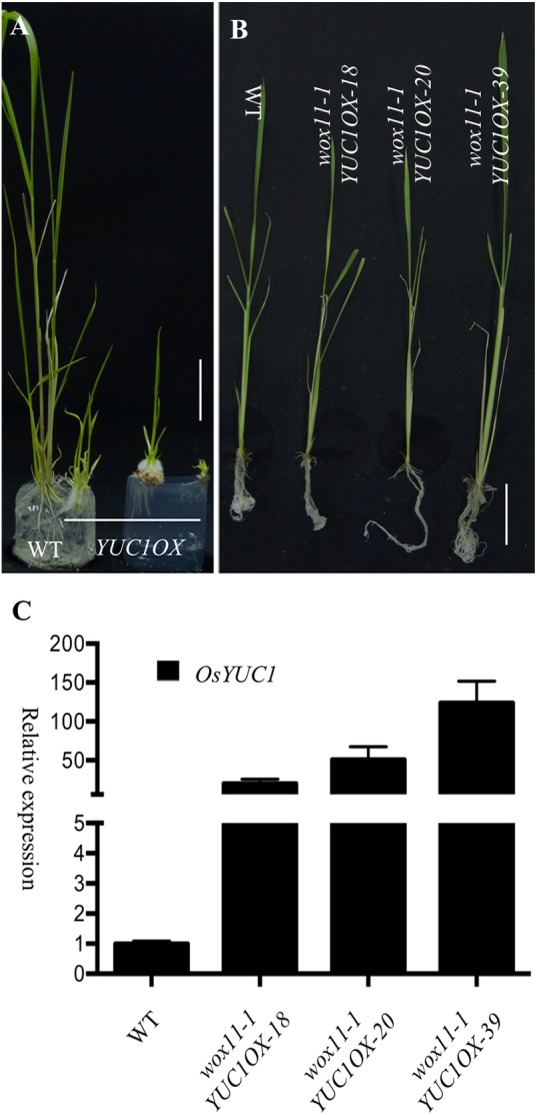
The crown root proliferation in *OsYUC1* overexpression lines is dependent on the presence of *WOX11*. **(A)** Overexpression of *OsYUC1* in Hwa cultivar, which was the background of *wox11-1*, promoted root development. *YUC1OX* refers to *OsYUC1* overexpression. Bar = 3 cm. **(B)** Overexpression of *OsYUC1* in *wox11-1* mutant background was not able to induce crown root development. No ectopic crown roots were observed in *OsYUC1* overexpression lines. *wox11-1OsYUC1OX-18*, *wox11-1OsYUC1OX-20*, and *wox11-1OsYUC1OX-39* were three independent T0 plants. Bar = 4 cm. **(C)** Relative transcript levels of *OsYUC1* in *wox11-1* mutant lines. *wox11-1OsYUC1OX-18*, *wox11-1OsYUC1OX-20*, and *wox11-1OsYUC1OX-39* had much elevated *OsYUC1* transcripts levels.

If the root phenotypes of the *OsYUC* overexpression lines are mediated by WOX11, disruption of *WOX11* in the *OsYUC* overexpression lines would suppress the root proliferation phenotypes. We introduced our *OsYUC1* overexpression construct (*UBIQUITIN:OsYUC1*), which was able to induce massive root development in wild type Zhonghua 11 (**Figure 1**), into the homozygous *wox11-1* mutant that is in the background Hwa. We found that *UBIQUITIN:OsYUC1* was able to cause the auxin overproduction phenotypes in Hwa as well (**Figure 6A** and Supplementary Figure S2). However, in the *wox11-1* mutants, the same construct did not cause the auxin overproduction phenotypes (**Figures 6B,C** and Supplementary Figure S2). We analyzed more than 120 independent *UBIQUITIN:OsYUC1 wox11-1* T0 plants and did not observe any obvious auxin overproduction phenotypes. We analyzed the expression levels of *OsYUC1* in the *UBIQUITIN:OsYUC1 wox11-1* and indeed the *OsYUC1* expression levels were increased. Our results clearly demonstrated that the root development phenotypes of *OsYUC* overexpression lines are dependent on the presence of *WOX11* and that *WOX11* is likely downstream of *OsYUC* genes.

### Overexpression *OsWOX11* Suppresses Crown Root Defects in *taa1/fib1* Mutant

**FIGURE 7:**
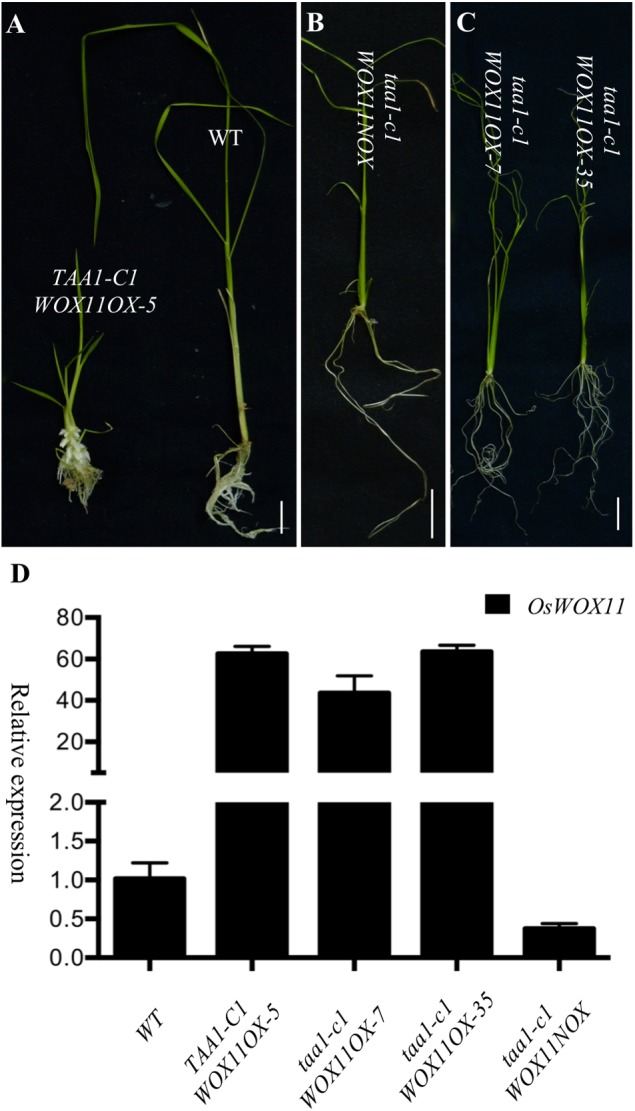
Rescue crown root defects in *taa1-c1* by overexpressing *OsWOX11.* **(A)** Overexpression of *WOX11* stimulates ectopic crown root development (left plant) compared to WT (right). *OsWOX11* refers to *OsWOX11* overexpression under the control of the strong maize *UBIQVITIN* promoter. Very few crown roots were developed in *taa1-c1* mutants **(B)**. But overexpression of *WOX11* stimulates crown root development in *taal* mutant background **(C)**. **(D)** The transcript levels (fold) of *OsWOX11* in the transgenic plants.

To further investigate whether *OsWOX11* functions downstream of YUC/TAA pathway, we overexpressed *OsWOX11* in *taa1/fib1*, which lacked the capacity to develop crown roots. Because homozygous *taa1/fib1* mutants are sterile, we used the seeds from heterozygous *taa1/fib1* plants to produce calli for transformation. T0 plants were genotyped to determine the zygosity of *taa1/fib1* mutation. Overexpression of *OsWOX11* in WT or heterozygous *taa1/fib1* produced ectopic crown roots as previously reported (**Figure 7** and Supplementary Figure S3). Interestingly, overexpression of *OsWOX11* in the *taa1/fib1* background also showed phenotypes similar to those observed in the *OsYUC* overexpression lines (**Figures 7A** left, 7C and Supplementary Figure S3). Our observation that overexpression of *OsWOX11* stimulated crown root development in *taa1/fib1* mutant strongly indicates that *WOX11* functions downstream of TAA/YUC in crown root development.

## Discussion

Our genetic analysis of *OsYUC* overexpression lines and the *taa1/fib1* mutants demonstrated that auxin synthesized by the TAA/YUC pathway is necessary and sufficient for crown root development in rice. We further showed that auxin-induced crown root initiation and elongation are mediated by the transcription factor WOX11, establishing a *YUC-Auxin-WOX11* module for crown root development in rice.

Auxin is the primary hormone that controls root development. In tissue culture, it is well known that high auxin/cytokinin ratio is necessary to stimulate root growth. Recently, it was shown that the cell fate transition during de novo root organogenesis in Arabidopsis requires YUC-mediated auxin biosynthesis (Chen et al., 2016). On the other hand, Arabidopsis auxin overproduction mutants superroot1 (Mikkelsen et al., 2004), superroot2 (Pacurar et al., 2014) and auxin overproduction transgenic lines including iaaM overexpression lines (Cheng et al., 2006), CYP79B2 overexpression lines (Zhao et al., 2002) and YUC overexpression lines (Zhao et al., 2001) all have root phenotypes. SUR1 and SUR2 produce adventitious roots from hypocotyls whereas iaaM, CYP79B2, and YUC overexpression lines have shorter primary roots and more root hairs (Zhao et al., 2001; Zhao et al., 2002). Auxin overproduction in rice by overexpressing OsYUC genes also promotes adventitious/crown root development and produces more root hairs, indicating that auxin is sufficient for the development of adventitious roots in both rice and Arabidopsis (Yamamoto et al., 2007). In Arabidopsis, mutations in TAAs or YUCs can completely eliminate root development or greatly reduce root elongation and lateral root development (Cheng et al., 2007; Stepanova et al., 2008). In rice, the *taa1/fib1* fails to make crown roots and produced fewer lateral roots (Yoshikawa et al., 2014). It is clear that the TAA/YUC biosynthesis pathway is conserved between Arabidopsis and rice. Moreover, the roles of auxin produced by TAA/YUC in root development are also very similar in Arabidopsis and rice.

*WOX11*, a *WUSCHEL* (*WUS*)-related Homeobox (*WOX*) gene, is sufficient to stimulate crown root development in rice (Zhao et al., 2009). Interestingly, the functions of *WOX11* in root development appeared to be conserved between Arabidopsis and rice as well. WOX11 along with its close homolog WOX12 controls the first-step cell fate transition during *de novo* root organogenesis in Arabidopsis (Liu et al., 2014). Much is known about how WOX11 regulates root development. OsWOX11 physically interacts with the ERF3 and regulates the cytokinin-responsive gene *RR2*, which plays a role in crown root development (Zhao Y. et al., 2015). Interestingly, CRL5 is also an ERF protein and also regulates cytokinin signaling (Kitomi et al., 2011). It will be interesting to test whether CRL5 and WOX11 physically interact with each other and whether ERF3 and CRL5 have overlapping functions. AtWOX11 was shown to physically interact with LBD16 and the WOX11-LBD16 was shown to promote the root primordium-like identity during Arabidopsis tissue culture. Shoot regeneration needs suppression of LBD16 expression in Arabidopsis (Liu et al., 2018). Interestingly, the *crl1*, which encodes the OsLBD3-2, fails to produce any crown roots (Inukai et al., 2001; Coudert et al., 2015), suggesting that CRL1 may also interact with WOX11 and the OsLBD3-2-WOX11 may have functions in rice similar to those of WOX11-LBD16 in Arabidopsis. Comparison of transcription of *wox11* and WT in conjugation of WOX11 binding sites have revealed that WOX11 target genes, which are mainly involved in cytokinin homeostasis/signaling, stress response, and redox metabolic processes (Jiang et al., 2017).

Several previous observations led us to connect WOX11 to auxin synthesized by the TAA/YUC pathway. First, both YUC-mediated auxin biosynthesis and WOX11 are required for cell fate transition occurring during de novo root organogenesis in Arabidopsis (Chen et al., 2016). Second, WOX11 is induced by auxin treatment in Arabidopsis (Liu et al., 2014). More importantly, overexpression of OsYUC1 produced phenotypes similar to those of WOX11 overexpression lines. In this work, we established that WOX11 is required for auxin-mediated crown root development. Moreover, we demonstrated that WOX11 functions downstream of auxin produced by TAA/YUC.

There is still a missing link between auxin produced by YUC/TAA and WOX11. We hypothesized that auxin triggers the degradation of AUX/IAA repressors, subsequently ARFs can activate downstream signaling components including WOX11. This hypothesis is consistent with previous findings that dominant IAA mutants severely affected crown root development (Jun et al., 2011; Kitomi et al., 2012; Zhu et al., 2012). We identified four putative ARF binding sites (TGTCTC/ACAGAG) in the OsWOX11 promoter region (Supplementary Table S1), providing a potential mechanism for auxin to regulate WOX11 expression through ARFs. It will be interesting to find which ARF may bind to the auxin response elements in WOX11 promoter region. Based on our genetic analysis, we propose that developmental and environmental signals activate the TAA/YUC auxin biosynthesis pathway to produce auxin, which subsequently triggers a signal transduction pathway to activate WOX11 expression and root development.

## Accession Numbers

Sequence data of rice genes in this article are accessible in the GenBank/EMBL data libraries with the following accession numbers: *OsYUC1*, LOC_Os01g45760; *OsYUC2*, LOC_Os05g45240; *OsYUC3*, LOC_Os01g53200; *OsYUC4*, LOC_Os01g12490; *OsYUC5*, LOC_Os12g32750; *OsYUC6*, LOC_Os07g25540; *OsYUC7*, LOC_Os04g03980; *OsYUC8*, LOC_Os03g06654; *OsYUC9*, LOC_Os01g16714; *OsYUC10*, LOC_Os01g16750; *OsYUC11*, LOC_Os12g08780; *OsYUC12*, LOC_Os02g17230; *OsYUC13*, LOC_Os11g10140; *OsYUC14*, LOC_Os11g10170; *OsTAA1*, LOC_Os01g07500; *OsWOX11*, LOC_Os07g48560.

Sequence data of Arabidopsis genes can be found in the GenBank/EMBL data libraries using the following accession numbers: *YUCCA1*, At4g32540; *YUCCA2*, At4g13260; *YUCCA3*, At1g04610; *YUCCA4*, At5g11320; *YUCCA5*, At5g43890; *YUCCA6*, At5g25620; *YUCCA7*, At2g33230; *YUCCA8*, At4g04610; *YUCCA9*, At1g04180; *YUCCA10*, At1g48910; *YUCCA11*, At1g21430.

## Author Contributions

TZ and YZ conceived the study and designed the experiments. TZ, RL, JX, LY, and RW performed the experiments. TZ and YZ wrote the manuscript.

## Conflict of Interest Statement

The authors declare that the research was conducted in the absence of any commercial or financial relationships that could be construed as a potential conflict of interest.
