# Supplementary material for: The YUCCA-Auxin-WOX11 Module Controls Crown Root Development in Rice
Source: Front Plant Sci. 2018 Apr 23;9:523. doi: 10.3389/fpls.2018.00523 (PMC5925970; doi:10.3389/fpls.2018.00523)
Supplement: Supplementary file 1 [file Table_1.PDF]

Supplemental Table S1. Auxin related cis-element in *WOX11* promoter.

The minus sign "-" indicate the distance in base pairs from transcription start site. "Reverse" means the motif is located on the complementary strand. The motifs were retrieved from the PLACE website (http://www.dna.affrc.go.jp/PLACE/).

Cis-element	Sequence	Site	Function
**ARFAT**	TGTCTC	2214 (Reverse)	ARF (auxin response factor) binding site
**ARFAT**	TGTCTC	4903 (Reverse)	ARF (auxin response factor) binding site
**ARFAT**	TGTCTC	-6899	ARF (auxin response factor) binding site
**ARFAT**	TGTCTC	-4356	ARF (auxin response factor) binding site
