# Supplementary material for: The YUCCA-Auxin-WOX11 Module Controls Crown Root Development in Rice
Source: Front Plant Sci. 2018 Apr 23;9:523. doi: 10.3389/fpls.2018.00523 (PMC5925970; doi:10.3389/fpls.2018.00523)
Supplement: Supplementary file 2 [file Table_2.PDF]

Supplemental Table 2: Primers used in the study.

Experiment	Primer name	Primer sequence (5'-3')
wox11-1 mutant identification	F2	GGCTGATACTAGCTAGGACACTGA
	R2	CTCAACTCGATCAAGACGACCT
	L2	CTAGAGTCGAGAATTCAGTACA
Real-time PCR analysis of different overexpression lines	Actin-RTF	TGGCATCTCTCAGCACATTCC
	Actin-RTR	TGCACAATGGATGGGCCAGA
	WOX11-RTF	CCAGATGGGCGAGAGCTACT
	WOX11-RTR	CGTTGCCATCGATCAATCAA
	YUC1-RTF	TCATCGGACGCCCTCAACGTCGC
	YUC1-RTR	GGCAGAGCAAGATTATCAGTC
Vectors construction	WOX11OE-F	TTACGAACGATAGCCGGTACGCGAGCTCTAGGTGTTCGAC
	WOX11OE-R	TCTAGAGGATCCCCGGGTACATCGACGAATCGCTCAACTC
	YUC1OE-F	TTACGAACGATAGCCGGTACACCTAGTTGGCAGTAAAAAAAGC
	YUC1OE-R	TCTAGAGGATCCCCGGGTACGATGCTCAATAGGCAAGTAA
	YUC3OE-F	TTACGAACGATAGCCGGTACTCACCAAGTCTTCTGGCGATAC
	YUC3OE-R	TCTAGAGGATCCCCGGGTACTTACTCCACCGAGGATGATACA
	YUC5OE-F	TTACGAACGATAGCCGGTACCATGCCAACCTCGCAACAAG
	YUC5OE-R	TCTAGAGGATCCCCGGGTACTCATTCTCGACCAGCAGACG
	YUC6OE-F	TTACGAACGATAGCCGGTACCCCTTCACTCCCTTCACCACC
	YUC6OE-R	TCTAGAGGATCCCCGGGTACTTGCGTCTCATCATCATCACCTC
	YUC7OE-F	TTACGAACGATAGCCGGTACGCTCACCTCCTATCCACCAACAC
	YUC7OE-R	TCTAGAGGATCCCCGGGTACTCCATCAACTGGCAAGCTCACT
	YUC8OE-F	TTACGAACGATAGCCGGTACCTTAGCTCATCGGTTCTTGGG
	YUC8OE-R	TCTAGAGGATCCCCGGGTACTGCACTCTTTCAGCCGTATCTG
	YUC10OE-F	TTACGAACGATAGCCGGTACCCTCCAACGCGACTCCCTTAT
	YUC10OE-R	TCTAGAGGATCCCCGGGTACTCACAGACCATTAAGGCCAAACTA
	YUC11OE-F	TTACGAACGATAGCCGGTACTTCACTTCCTTCCCTTTCCT
	YUC11OE-R	TCTAGAGGATCCCCGGGTACTCCAAACAAAGGGCTTCACA
	YUC14OE-F	TTACGAACGATAGCCGGTACATACAGGCACGTCCATTCTCC
	YUC14OE-R	TCTAGAGGATCCCCGGGTACCACAGAGAGACGAGCCCTTTT
	OsU3-F	CCCCTTTCGCCAGGGGTACCGTAATTCATCCAGGTCTCCAAG
	OsU3-R	TACGAATTCGAGCTCGGTACCGCTGTGCCGTACGACGGTACG
	TAA1C1-F	AGAACTCCTCGAACATGGTCGTTTTAGAGCTAGAAATAGCAAGTTA
	TAA1C1-R	GACCATGTTCGAGGAGTTCTGCCACGGATCATCTGCACAACTC
	TAA1C2-F	AGGTTCCAAGCGAGCAACGGGTTTTAGAGCTAGAAATAGCAAGTTA
	TAA1C2-R	CCGTTGCTCGCTTGGAACCTGCCACGGATCATCTGCACAACTC
taa1/fib1 sequence	Taa1-seqF	AAGTTGCCATGGGTGGATGT
	Taa1-seqR	TTTTTGGACGGAGGGAGTAAT
Transgenic	CAS9F	GCATGAAGAGGATCGAGGAG

identity	CAS9R	GATCTCTTGCTCGGACTTGG
