# Supplementary material for: The YUCCA-Auxin-WOX11 Module Controls Crown Root Development in Rice
Source: Front Plant Sci. 2018 Apr 23;9:523. doi: 10.3389/fpls.2018.00523 (PMC5925970; doi:10.3389/fpls.2018.00523)
Supplement: Supplementary file 3 [file Image_1.PDF]

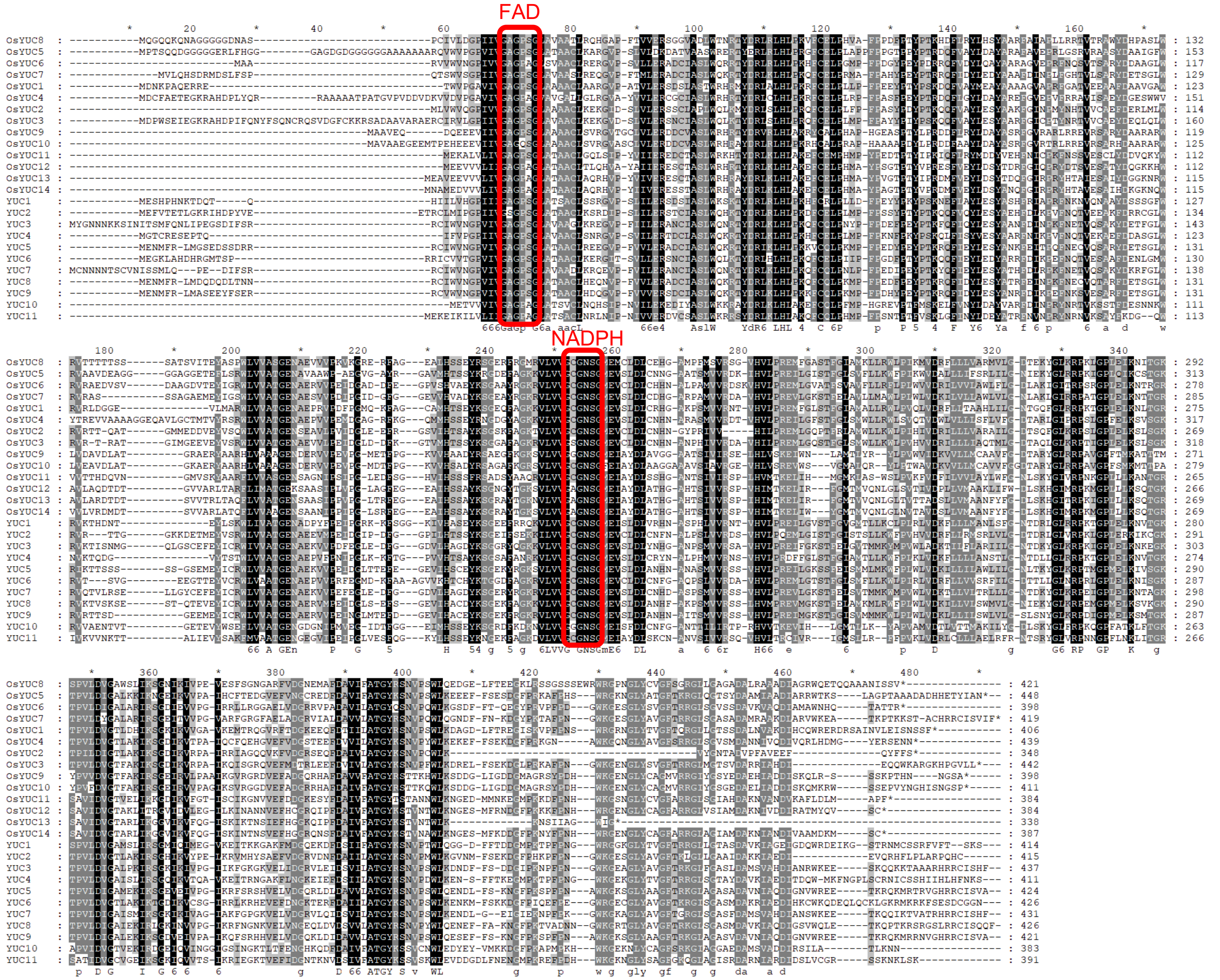

Supplemental Figure S1. Alignment of rice and Arabidopsis YUCCAs proteins.
Black and gray boxes indicate identical and similar amino acids, respectively. The putative FAD (GAGPSG) and NADPH (GCGNSG) binding motifs are boxed.
