# Supplementary material for: The YUCCA-Auxin-WOX11 Module Controls Crown Root Development in Rice
Source: Front Plant Sci. 2018 Apr 23;9:523. doi: 10.3389/fpls.2018.00523 (PMC5925970; doi:10.3389/fpls.2018.00523)
Supplement: Supplementary file 4 [file Image_2.PDF]

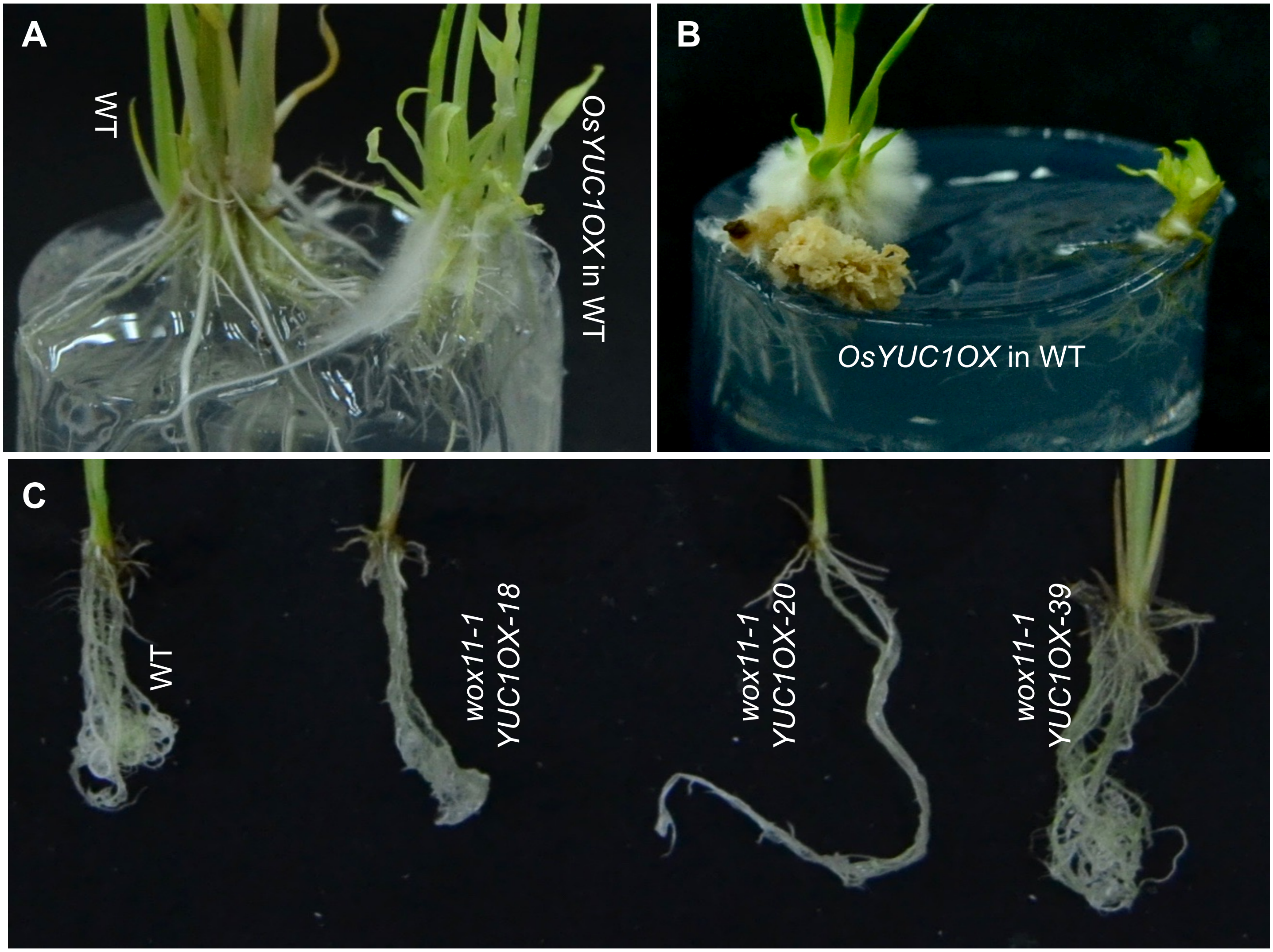

Figure S2. Suppression of the crown root over-proliferation phenotype of *OsYUC1* overexpression lines by disrupting *WOX11*. (A-B) The close-up images of Fig 6A, B. Overexpression of YUC1 stimulated crown root and root hair development. C) The OsYUC1 overexpression phenotypes disappeared in *wox11* mutant background.

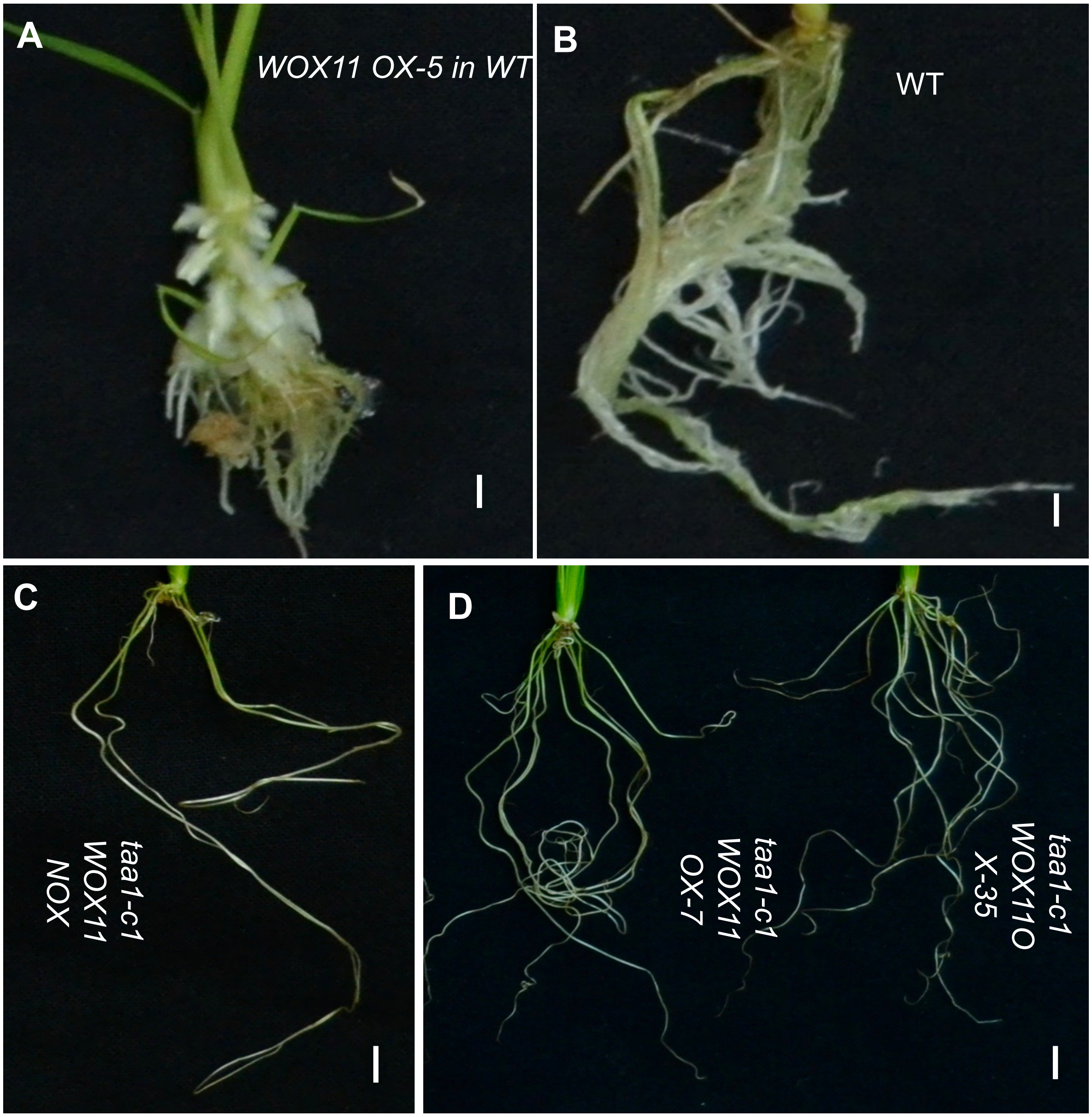

Figure S3. Crown roots phenotype of *OsWOX11* overexpression lines is dependent on the presence of the TAA/YUC auxin biosynthetic pathway. The figure shows the close-up images of Fig 7A, B,C and D respectively. *WOX11 OX-5* refers to the *OsWOX11* overexpression line 5. The taa1-c1 refers to a *crispr* mutant of *taa1*. *NOX* means NOT Overexpressed. *OX* means overexpression. Bar=1cm.
